# FORM: An Australian method for formulating and grading recommendations in evidence-based clinical guidelines

**DOI:** 10.1186/1471-2288-11-23

**Published:** 2011-02-28

**Authors:** Susan Hillier, Karen Grimmer-Somers, Tracy Merlin, Philippa Middleton, Janet Salisbury, Rebecca Tooher, Adele Weston

**Affiliations:** 1 international Centre for Allied Health Evidence, University of South Australia, Australia; 2 Adelaide Health Technology Assessment (AHTA), School of Population Health and Clinical Practice, University of Adelaide, Australia; 3 Australian Research Centre for Health of Women and Babies, University of Adelaide, Australia; 4 Biotext Pty Ltd, PO Box 178, Deakin West, ACT 2600, Australia; 5 Vaccinology and Immunology Research Trials Unit, Women's and Children's Hospital, Australia; 6 Health Technology Analysts Pty Ltd, Australia

## Abstract

**Background:**

Clinical practice guidelines are an important element of evidence-based practice. Considering an often complicated body of evidence can be problematic for guideline developers, who in the past may have resorted to using levels of evidence of individual studies as a quasi-indicator for the strength of a recommendation. This paper reports on the production and trial of a methodology and associated processes to assist Australian guideline developers in considering a body of evidence and grading the resulting guideline recommendations.

**Methods:**

In recognition of the complexities of clinical guidelines and the multiple factors that influence choice in health care, a working group of experienced guideline consultants was formed under the auspices of the Australian National Health and Medical Research Council (NHMRC) to produce and pilot a framework to formulate and grade guideline recommendations. Consultation with national and international experts and extensive piloting informed the process.

**Results:**

The FORM framework consists of five components (evidence base, consistency, clinical impact, generalisability and applicability) which are used by guideline developers to structure their decisions on how to convey the strength of a recommendation through wording and grading via a considered judgement form. In parallel (but separate from the grading process) guideline developers are asked to consider implementation implications for each recommendation.

**Conclusions:**

The framework has now been widely adopted by Australian guideline developers who find it to be a logical and intuitive way to formulate and grade recommendations in clinical practice guidelines.

## Background

Best practice in health care should be guided by the results of research on the safety and effectiveness of different courses of clinical action. This evidence needs to be assembled, justified and presented in the form of health advice for multiple stakeholders including health professionals, decision makers and consumers of health care. Clinical practice guidelines are recognised as one of the best ways to present recommended courses of action based on research evidence, although recommendations are often presented inconsistently [1]. Where such evidence is not available, guidelines may use consensus-based practice points and/or identify areas requiring further research. Both format and content can adversely affect the adoption and integration of guidelines into clinical practice [2].

The National Health and Medical Research Council (NHMRC) of Australia has been a world leader in developing and supporting the development of evidence-based health advice, including clinical practice guidelines. As early as 1999, the NHMRC commissioned and published 'Guidelines for Guideline Development' [3], anticipating the need for a comprehensive set of resources to help guideline developers produce high quality guidelines. This was followed by a more detailed series of handbooks on different aspects of finding and reviewing clinical research [4].

Australian guideline developers must comply with NHMRC standards in order to gain NHMRC approval. These standards (such as rigorous evidence-based methods, multidisciplinary panels and public consultation processes) have resulted in NHMRC approved guidelines being of higher quality than those developed outside NHMRC processes [5].

By 2004, it had become clear that the NHMRC standards required expansion and revision in response to the rapid growth and diversification of clinical practice guidelines in Australia and elsewhere. There were two main areas where a need for revision was identified. The first was the need to develop a set of levels (or hierarchy) of evidence which would cover the different individual study designs used to address the different types of questions formulated by guideline development panels. This work (covering interventions, diagnostic accuracy, prognosis, aetiology and screening) is outlined in Merlin et al [6]. The second area was the need to develop a new system, or adaptation of an existing system, of formulating and grading recommendations for clinical practice guidelines that incorporated an assessment of the 'body of evidence'.

### The concept of a body of evidence

Many guideline recommendations have been rated solely according to the level of evidence of the individual studies contributing to that recommendation. In the late 1990 s and early 2000 s, NHMRC prepared a series of handbooks to assist clinical practice guideline developers. These handbooks stated that other elements such as study quality, size and precision of study results, and relevance to local practice were also important [3,4]. They did not, however, go as far as providing a transparent logical framework for assessing these elements when formulating recommendations. What was needed was a method for considering all of these elements across all of the research studies addressing the clinical question as a whole (the 'body of evidence') like some other guideline development methodologies (such as those used by the Scottish Intercollegiate Guidelines Network or the National Institute for Health and Clinical Excellence). Recommendations based on the body of evidence could then be graded according to the degree of confidence that implementing the suggested course of action would lead to improved patient health outcomes.

In recognition of this need, and in response to requests from methodological experts that consult for the NHMRC on guideline development (Guidelines Assessment Register [GAR] consultants) (see Appendix 1), the NHMRC undertook to revise and update its methodological approaches. This paper reports on the production and trial of a methodology and associated processes to assist Australian guideline developers in considering a body of evidence and grading the resulting guideline recommendations.

## Methods

In 2004, the NHMRC commissioned a review of existing frameworks for assessing evidence internationally [7]. This internal report provided a resource for a working party (comprising GAR consultants and NHMRC personnel - see Appendix 1 for members) to review existing practice, design and/or adapt a framework for grading a body of evidence and pilot this process with Australian guideline developers.

The report identified nine possible systems for use in developing clinical practice guidelines. Of these, three were considered to be most useful for informing the development of an Australian guideline recommendation process. These frameworks were the Scottish Intercollegiate Guidelines Network (SIGN) system and considered judgement statement (SIGN50, revised 2008)[8]; the Strength of Recommendation Taxonomy (SORT) [9]; and the Grading of Recommendations, Assessment, Development and Evaluation (GRADE) [10].

These systems were discussed at a face-to-face meeting of the working party with respect to their advantages and disadvantages and compatibility with the existing advice in the NHMRC 'Guidelines for Guideline Development' handbooks. A consensus was reached about how these frameworks could be adapted in the new process. From the three systems, we combined elements to achieve our objectives, which were: to have a system that matched and complemented the current NHMRC evidence dimensions and documents as closely as possible; simplicity and clarity of approach; and to provide transparent method/s of formulating and documenting judgments to give a graded set of recommendations. The working party drafted a new framework for grading recommendations and this was refined by extensive email consultation and iteration within the group.

The resulting draft framework was piloted by GAR consultants working with guideline developers between 2005 and 2009. There were five main methods to gather feedback:

• Known experts in the international guideline field were approached by NHMRC directly for comment on the draft system - this was a formal request and responses were semi-structured in that the experts were free to review in their own style,

• Key evidence-based assessment organisations in Australian and New Zealand were invited to register feedback on the website where the system was posted,

• All guideline development groups working within the NHMRC endorsement framework during this period used FORM under the guidance of the GARs - they were all invited to offer feedback during and after the process.

• The draft process was presented at key conferences and interactive workshops (eg International Cochrane Colloquium [11]),

• The website was open for the 5 years (passive seeking) and included a structured feedback form.

Following this initial period of consultation (up until 2007) the FORM's framework was further refined, taking account of the feedback received, and the public consultation period was extended to June 2009. During the development, trialing and refinement period from 2004 to 2009, the international guideline community continued to debate and evolve other systems of guideline production - these developments were monitored and helped to inform the Australian process. The revised version of FORM was subsequently endorsed by the Council of the NHMRC.

## Results

The new FORM framework was loosely based on the SIGN considered judgement form [8]. It provides guideline developers with a structured process for considering the whole body of evidence relevant to a particular clinical question, in the context of the setting in which it is to be applied. FORM recognises that ascribing a *level of evidence *to each study that reflects the risk of bias in its design, is only one small part of assessing evidence for a guideline recommendation. FORM provides a framework for assessing all the studies relevant for a recommendation against five criteria: the *evidence base *(i.e. number, level and risk of bias in included studies); the *consistency *of findings between studies; the *clinical impact *suggested by the evidence base; the *generalisability *of the results to the population for whom the guideline is intended; and the *applicability *of the results to the Australian (and/or local) health care setting. Under FORM, these five key components are individually assessed for each clinical question giving a picture of both the internal and external validity of the evidence base under consideration.

### Key components of FORM

#### 1. Evidence base

The evidence base is assessed in terms of the quantity and quality of the studies identified by a systematic literature review for the clinical question concerned ('included studies'). Study quality relates to an assessment of the risk of bias inherent in the conduct, design and reporting of results in the included studies. The guideline developers are free to choose the most relevant process or tool to assess risk of bias. To ensure that consideration is given to the full range of study designs required to assess the breadth of clinical questions in a guideline, the GAR consultants also developed levels of evidence to address different clinical questions (prognosis, diagnostic accuracy, aetiology etc). This has been comprehensively addressed by Merlin et al [6] (see also, NHMRC website: http://www.nhmrc.gov.au/guidelines/developers.htm)

#### 2. Consistency

The consistency component of the 'body of evidence' assesses the extent to which the findings are consistent across the included studies (including across a range of study populations and study designs). This allows users to assess whether the results are likely to be replicable or only likely to occur under certain conditions. Consistency may be assessed where appropriate as statistical heterogeneity (applying an I-squared statistic for example) or more likely will require the users to make a judgment about the overall direction of effects across multiple studies with reference to clinical heterogeneity. Possible sources of inconsistency (heterogeneity) in the results of studies may be differences in the study design, the quality of the studies (risk of bias), the population studied, and varying definitions of outcomes being assessed. Should results differ for certain subpopulations, this could then be reflected in the development of the recommendation.

#### 3. Clinical impact

Clinical impact is a measure of the likely benefit that application of the guideline would have across the target population, and involves a clinical judgement. Factors that need to be taken into account when estimating clinical impact include: the relevance of the evidence to the clinical question; the statistical precision and size (and clinical importance) of the effect reported in the evidence-base; the relevance of the effect to patients, compared to other management options; the duration of therapy required to achieve the effect; and the balance of risks and benefits to the patient group, including potential harm. A hypothetical example of incorporating both clinical importance and potential harm may be for the use of statins in the control of dyslipidaemia where there is a very large body of evidence with low risk of bias indicating *a substantial *reduction in risk of cardiovascular events. In this case a qualifying recommendation could be made to differentiate the small group of people who may experience adverse events as a result of statin therapy.

Clinical impact is arguably the most subjective of the five evidence components rated in the evidence statement. However, we have found in assisting many guideline development groups to produce clinical practice guidelines using the FORM process that it is often clearer for clinicians than it is for methodological experts. Clinicians seem to grasp the net benefit concept quite easily, although often robust discussions occur before a consensus is reached regarding the rating of this component. A strength of FORM is that these discussions contribute to formulating appropriate recommendations, and the final conclusion can be documented so that users of the guideline can see how the developers arrived at the recommendation.

#### 4. Generalisability

The assessment of generalisability involves determining how precisely the available body of evidence answers the clinical question that was asked. Issues to be considered include: how well the participants and settings of the included studies match the patient population being targeted by the guideline; the clinical setting where the recommendation will be implemented; and other factors such as the stage of the disease (e.g. early versus advanced), the duration of illness and (for diagnostic accuracy questions) the prevalence of the disease in the study population as compared to the target population for the guideline.

#### 5. Applicability

This component addresses whether the evidence base is relevant to the Australian health care system generally, or to more local settings for specific recommendations (such as rural areas or cities). Factors that may reduce the direct application of study findings to the Australian or more local settings include organisational factors (e.g. availability of trained staff, clinic time, specialised equipment, tests or other resources) and cultural factors (e.g. attitudes to health issues, including those that may affect compliance with the recommendation).

### The FORM Matrix and Evidence Statement Form

The FORM matrix forms part of the overall process which is detailed in Additional file 1. Each of the components in the FORM matrix can be rated from A to D. The body of evidence supporting a recommendation rarely consists of the same rating for each of the five components. There may be a large number of studies with a low risk of bias and consistent findings, but which have only a limited clinical impact, and are not directly generalisable to the target population or applicable to the local (e.g. Australian) healthcare context. Alternatively, a body of evidence may consist of one or two randomised trials with small sample sizes that have a moderate risk of bias but have a very large clinical impact and are directly applicable to the local healthcare context and target population. By rating each of the five components separately, FORM allows for this mixture of components, while still reflecting the overall body of evidence supporting a guideline recommendation. The FORM Matrix provides guidance for users about how to rate each component of the body of evidence (see Table 1). The accompanying Evidence Statement Form is provided for guideline developers to complete for each clinical question with room for additional information and dissenting opinions to be recorded. A recommendation to answer the clinical question is developed in two stages. First, a rating is assigned for each of the five components described above and an evidence statement is written in passive voice to reflect the findings of the evidence base. Second, an overall recommendation or action statement is developed on the basis of the evidence statement and an overall grade is assigned to this recommendation that reflects the level of confidence in the evidence supporting the recommendation.

**Table 1:** NHMRC Body of evidence matrix

Component	A Excellent	B Good	C Satisfactory	D Poor
Evidence base^1^	One or more level I studies with a low risk of bias or several level II studies with a low risk of bias	One or two level II studies with a low risk of bias or an SR/several level III studies with a low risk of bias	One or two level III studies with a low risk of bias, or level I or II studies with a moderate risk of bias	Level IV studies, or level 1 to II studies/SRs with a high risk of bias

Consistency^2^	All studies consistent	Most studies consistent and inconsistency may be explained	Some inconsistency reflecting genuine uncertainty around clinical question	Evidence is consistent

Clinical impact	Very large	Substantial	Moderate	Slight or restricted

Generalisability	Population/s studied in body of evidence are the same as the target population in the guideline	Population/s studied in the body of evidence are similar to the target population for the guideline	Population/s studied in the body of evidence differ to the target population guideline but it is clinically sensible to apply this evidence to the target population^3^	Population/s studied in the body of evidence differ to the target population and hard to judge whether it is sensible to generalize to target population

Applicability	Directly applicable to Australian healthcare context	Applicable to Australian health care context with few caveats	Probably applicable Australian healthcare context with some caveats	Not applicable to Australian healthcare context

SR = systematic review; several = more than two studies

^1 ^Level of evidence determined from the NHMRC Evidence Hierarchy

^2 ^If there is only one study, rank this component as 'not applicable'

^3 ^For example, results in adults that are clinically sensible to apply to children OR psychosocial outcomes for one cancer that may be applicable to patients with another cancer

Evidence statements may be developed by outcome measures for each intervention and then the multiple evidence statements for a single question can be collapsed into a single recommendation. Guideline developers can produce a combined recommendation taking into account the balance of benefits and harms or separate recommendations for benefits and harms, if this is more appropriate. The FORM process allows considerable flexibility in developing the recommendation.

The overall grades for recommendations should indicate the strength of the body of evidence underpinning the recommendation. This assists users of the clinical practice guidelines to make appropriate and informed clinical judgments. Grade A or B recommendations are generally based on a body of evidence that can be trusted to guide clinical practice, whereas Grade C or D recommendations must be applied carefully to individual clinical and organisational circumstances and should be interpreted with caution (see Table 2). A recommendation cannot be graded A or B unless the evidence base and consistency of the evidence are both rated A or B. In some cases, lower-graded evidence statements may not provide sufficient confidence to support an evidence-based recommendation at all. However, the framework allows *Good Practice Points (GPP) *to be included when developers feel it is important to provide non-evidence-based guidance.

**Table 2:** Definition of NHMRC grades of recommendations

Grade of recommendation	Description
A	Body of evidence can be trusted to guide practice

B	Body of evidence can be trusted to guide practice in most situations

C	Body of evidence provides some support for recommendation(s) but care should be taken in its application

D	Body of evidence is weak and recommendation must be applied with caution

In formulating the recommendation users are advised to address the specific clinical question and to use action statements. The wording of the recommendation should reflect the strength of the body of evidence. Words such as 'must' or 'should' or 'use' are included when the evidence underpinning the recommendation is strong, and words such as 'might' or 'could' or 'consider' are used when the evidence base is weaker.

The following recommendations illustrate these points and are taken from the NHMRC Clinical Practice Guidelines for the Management of Melanoma in Australia and New Zealand (NHMRC 2008). These show that the evidence base, consistency and impact were high for dermoscopy, but not so high for total body photography (also indicated by the use of the verb 'recommended' in the first case and 'consider' in the second):

• Training and utilisation of dermoscopy is recommended for clinicians routinely examining pigmented skin lesions: Grade A;

• Consider the use of baseline total body photography as a tool for the early detection of melanoma in patients who are at high risk for developing primary melanoma: Grade C (p xxii [12]).

Developers are also asked to consider how the guideline will be implemented at the time that the guideline recommendations are being formulated. The Evidence Statement Form requests developers to consider whether: the recommendation will result in changes in usual care; there are any resource implications associated with implementing the recommendation; the implementation of the recommendation will require changes in the way care is currently organised; and the guideline development group are aware of any barriers to the implementation of the recommendation. This information is used to inform the implementation plan for the Guideline.

### Feedback, piloting and users' experiences

Over the trial and consultancy period for the FORM grading process, we obtained feedback from invited experts (see acknowledgements), from current guideline developers and from the public. These issues and suggestions were carefully considered at the face-to-face meeting of the GAR consultants in 2007 (see methods). Where appropriate, we amended the FORM methodology and/or supporting documents to incorporate the suggestions or address problems. This iterative process ensured that the development of FORM was responsive to the needs of its core user group - guideline developers - and was as clear and comprehensible as possible, even for developers with limited methodological expertise. It also allowed the FORM development process to keep abreast of the sometimes rapidly changing methodology underpinning guideline development internationally and incorporate changes into FORM as appropriate. As developers of FORM and also methodological experts assisting guideline developers we (the authors) have been able to field-test the FORM process and gain first-hand feedback and direct experience about problems and issues encountered. This has been invaluable in modifying FORM to be more effective and useful.

The following issues were identified in the first consultation and addressed in the second iteration of FORM where appropriate:

• deciding between grades - but this has become easier with time and familiarity

• determining and extracting relevant information from synthesised sources (such as existing systematic reviews) which are incompletely reported

• insufficient funding, human resources and/or time for the rigorous systematic literature reviews needed to underpin the evidence statements

• need to accommodate subjectivity in the interpretation of the components and the final recommendation/s

In response to specific suggestions made in the first consultation period, we made the following modifications to the FORM supporting documentation:

• revision of the notes, matrix and form to be more user friendly

• the addition of 'explanatory notes' sections for developers to document reasons for particular decisions within the matrix

• the addition of a 'dissenting opinions' and 'unresolved issues' sections to the Evidence Statement Form to keep decision making transparent and informed

• a flowchart to assist in navigation

Feedback from the second stage of consultation showed that the modifications were a major improvement and that guideline developers agreed that the FORM system of grading was an improvement on the previous system where recommendations were 'graded' according to the level of evidence from the NHMRC evidence hierarchy [3,6]. They also reported that the framework offers an opportunity to develop guidelines that improve dissemination and uptake in clinical practice. With increasing familiarity users have found the framework fairly simple to use.

As methodological experts assisting guideline developers, we have found the framework provides additional flexibility, especially when handling evidence with more than one outcome measure (for example overall survival, pain, readmission rates). Variable results/evidence statements for multiple outcomes can be captured by a single recommendation. Furthermore, the framework also allows a recommendation to be developed that balances the benefits and harms of an intervention (i.e. safety and effectiveness), but with enough flexibility to keep them separate if it is felt to be important. More than 20 NHMRC guidelines have now been completed using FORM.

## Discussion

The formulation and inclusion of recommendations is one of the defining differences between clinical practice guidelines and other evidence syntheses such as systematic reviews. A recent review of the adequacy of guideline recommendations has highlighted that over half of the recommendations (52.7%) give no indication of the strength of that recommendation [1].

The FORM process for formulation and grading of recommendations in clinical practice guidelines is logical, simple to use and intuitive. Its concurrent development with Australian levels of evidence [6] means that NHMRC can provide Australian (and other) guideline developers with an integrated framework for producing high-quality recommendations that represent best-practice and are implementable, acceptable and appropriate for the local health care system. The framework is also generic - the same processes can be used to formulate and grade recommendations for any type of clinical question, despite the differences in the type of evidence required to address that question (e.g. questions of diagnostic test accuracy, risk factors for disease progression or poor prognosis). Furthermore, health service providers can implement the evidence-based course of action with appropriate modification in light of the individual patient's values and preferences.

In areas like public health where there may never be high-level evidence supporting the use of different interventions, practice recommendations developed using other grading systems would consistently rate a lower grade than is felt appropriate by experts in those fields. Examples of such areas include large-scale dietary questions, passive smoking or exposure to environmental chemicals. This does not occur using the FORM methodology. Using the NHMRC levels of evidence for aetiology questions as an alternative to the levels for intervention questions [6] allows the evidence base component of our grading system to be rated higher than would otherwise occur and this would be reflected in the overall grade of recommendation.

The extensive pilot of FORM and subsequent uptake by both new and experienced guideline developers has shown that the framework is feasible and accepted. The component approach allows transparency in how recommendations are formulated, with users of the guidelines able to explicitly see the various contributions of factors such as quality of the evidence and clinical impact. A further strength is that implementation and resourcing issues are considered separately, which means that effective but potentially costly interventions are not penalised with a downgraded recommendation as the developers of this system felt that users' willingness to pay will vary according to the context of use. Arguably the greater ability to differentiate strength of recommendation (four levels) in FORM offers more precision for developers.

### Limitations

The UK National Institute for Health and Clinical Excellence (NICE) has decided to discontinue summary grades for recommendations, on the grounds that their previous grading system was being misinterpreted. They have stated that they are not sure that the GRADE system's approach to summary labels overcomes this [13]. We are not aware of this sort of misinterpretation occurring with FORM, and believe that the benefits of grading outweigh the harms as clinicians are striving for clear-cut health advice to assist with their individual decision-making. However, ongoing monitoring and periodic review of the application and use of FORM needs to be considered.

Recommendation formulation and grading can be particularly challenging when the evidence is scant and/or poor, or conflicting. NICE has outlined some strategies to address these challenges, including using consensus when no evidence is found for a particular clinical question and highlighting gaps in the evidence where evidence is scant or poor.[14] NICE reminds us that whenever guidelines are unable to rely on a solid evidence base other methods used for formulating recommendations must be transparent and set out clearly in the guideline. A particular strength of an explicit process such as FORM is that the path from evidence to recommendation is made clear.

Current evidence frameworks are grappling with how to integrate other forms of evidence needed to answer qualitative questions such as optimal quality of life, and we anticipate that FORM will need to be periodically reassessed in the light of international debate about levels of evidence and grading recommendations.

The purpose of clinical practice guidelines is to change or guide health professionals' behaviour and to improve quality of care. Therefore, the ultimate test of guidelines and the processes used to develop and implement guidelines will be improved health outcomes and improved systems. One way of facilitating this is by developing recommendations that are transparently produced through a process that is user-friendly, weighs up multiple concepts when formulating a course of action (much as the clinician does for an individual patient), and provides clear advice on the confidence or uncertainty associated with the recommended course of action.

## Conclusion

FORM provides a contemporary and internationally relevant structure within which clinical guideline developers can consider current literature related to specific clinical questions. It has been developed through a unique partnership of government, academic, private consultancy and clinical personnel with considerable experience in evidence-based practice and development of clinical practice guidelines. Our work with over 20 guideline developers during the piloting of the FORM process has demonstrated it to be a logical, simple to use and intuitive system for formulating and grading recommendations in clinical practice guidelines.

## Competing interests

The authors declare that they have no competing interest. Meeting attendance fees were paid to the authors (or their institutions) by the NHMRC - a not-for-profit research organisation funded by the Australian Government.

## Authors' contributions

All authors were involved in the process of drafting and revising the grading process as described and all authors were involved in the preparation and final approval of the current manuscript.

## Appendix 1

History of NHMRC Guidelines Assessment Register (GAR) and members of the Levels and Grades Working Party

In 2002, the NHMRC convened a register of methodological experts (Guidelines Assessment Register [GAR]) to assist external guideline developers in Australia through the process of identifying and synthesising evidence for guidelines in a way that complied with NHMRC specified requirements and would assist them in gaining NHMRC endorsement for their work. The main role of the GAR consultants was to oversee the methodological processes in external development of guidelines, particularly reviewing and classifying the quality of the evidence, and how these classifications correlated to the resultant recommendations. The expected outcome of the involvement of the GAR consultants was that consistently high quality guidelines would be submitted to HAC for approval, and that problems identified *post hoc *in guideline development could be pre-empted.

Kristina Coleman, Sarah Norris, Adele Weston (Health Technology Analysts Pty Ltd)

Karen Grimmer-Somers, Susan Hillier (iCentre for Allied Health Evidence, University of South Australia)

Tracy Merlin (Adelaide Health Technology Assessment, Discipline of Public Health, University of Adelaide)

Philippa Middleton, Rebecca Tooher (ARCH, University of Adelaide)

Janet Salisbury (Biotext Pty Ltd)

## Pre-publication history

The pre-publication history for this paper can be accessed here:

http://www.biomedcentral.com/1471-2288/11/23/prepub

## Supplementary Material

Additional file 1 **NHMRC Evidence Statement *(landscape Document)***. This file includes the FORM documents discussed - the matrix and trigger questions for guideline developers to complete the Evidence Statement. Click here for file
