# Supplementary material for: FORM: An Australian method for formulating and grading recommendations in evidence-based clinical guidelines
Source: BMC Med Res Methodol. 2011 Feb 28;11:23. doi: 10.1186/1471-2288-11-23 (PMC3053308; doi:10.1186/1471-2288-11-23)
Supplement: Additional file 1 — NHMRC Evidence Statement (landscape Document). This file includes the FORM documents discussed - the matrix and trigger questions for guideline developers to complete the Evidence Statement. [file 1471-2288-11-23-S1.DOC]

**Appendix 2:** **NHMRC Evidence Statement**

(If rating is not completely clear, use the space next to each criterion to note how the group came to a judgment.)

| **Key question(s):** | | | **Evidence table ref:** |
| --- | --- | --- | --- |
| **1. Evidence base** *(number of studies, level of evidence and risk of bias in the included studies)* | | | |
|  | A | **One or more level I studies with a low risk of bias or several level II studies with a low risk of bias** | |
|  | B | **One or two Level II studies with a low risk of bias or SR/several Level III studies with a low risk of bias** | |
|  | C | **One or two Level III studies with a low risk of bias or Level I or II studies with a moderate risk of bias** | |
|  | D | **Level IV studies or Level I to III studies/SRs with a high risk of bias** | |
| **2. Consistency** *(if only one study was available, rank this component as ‘not applicable’)* | | | |
|  | A | **All studies consistent** | |
|  | B | **Most studies consistent and inconsistency can be explained** | |
|  | C | **Some inconsistency, reflecting genuine uncertainty around question** | |
|  | D | **Evidence is inconsistent** | |
|  | NA | **Not applicable (one study only)** | |
| **3. Clinical impact** *(Indicate in the space below if the study results varied according to some unknown factor (not simply study quality or sample size) and thus the clinical impact of the intervention could not be determined)* | | | |
|  | A | **Very large** | |
|  | B | **Substantial** | |
|  | C | **Moderate** | |
|  | D | **Slight/Restricted** | |
| **4. Generalisability** *(How well does the body of evidence match the population and clinical settings being targeted by the Guideline?)* | | | |
|  | A | **Evidence directly generalisable to target population** | |
|  | B | **Evidence directly generalisable to target population with some caveats** | |
|  | C | **Evidence not directly generalisable to the target population but could be sensibly applied** | |
|  | D | **Evidence not directly generalisable to target population and hard to judge whether it is sensible to apply** | |
| **5. Applicability** *(Is the body of evidence relevant to the Australian healthcare context in terms of health services/delivery of care and cultural factors?)* | | | |
|  | A | **Evidence directly applicable to Australian healthcare context** | |
|  | B | **Evidence applicable to Australian healthcare context with few caveats** | |
|  | C | **Evidence probably applicable to Australian healthcare context with some caveats** | |
|  | D | **Evidence not applicable to Australian healthcare context** | |

| **Other factors** *(Indicate here any other factors that you took into account when assessing the evidence base (for example, issues that might cause the group to downgrade or upgrade the recommendation)* | | | | |
| --- | --- | --- | --- | --- |
|  | | | | |
| **EVIDENCE STATEMENT MATRIX**  *Please summarise the development group’s synthesis of the evidence relating to the key question, taking all the above factors into account.* | | | | |
| **Component** | **Rating** | **Description** | | |
| 1. Evidence base |  |  | | |
| 1. Consistency |  |  | | |
| 1. Clinical impact |  |  | | |
| 1. Generalisability |  |  | | |
| 1. Applicability |  |  | | |
| *Evidence statement*  *Indicate any dissenting opinions* | | | | |
| **RECOMMENDATION**  *What recommendation(s) does the guideline development group draw from this evidence? Use action statements where possible.* | | | **GRADE OF RECOMMENDATION** |  |
|  | | | | |
| **UNRESOLVED ISSUES**  *If needed, keep note of specific issues that arise when each recommendation is formulated and that require follow-up.* | | | | |
|  | | | | |

| **IMPLEMENTATION OF RECOMMENDATION**  *Please indicate yes or no to the following questions. Where the answer is yes please provide explanatory information about this. This information will be used to develop the implementation plan for the guidelines.* | |
| --- | --- |
| Will this recommendation result in changes in usual care? | **YES** |
|  | **NO** |
| Are there any resource implications associated with implementing this recommendation? | **YES** |
|  | **NO** |
| Will the implementation of this recommendation require changes in the way care is currently organised? | **YES** |
|  | **NO** |
| Are the guideline development group aware of any barriers to the implementation of this recommendation? | **YES** |
|  | **NO** |
